# Digital medicine and e-health in primary care. Selected abstracts from the virtual 90th EGPRN conference, 16–17 October 2020: All abstracts of the conference can be found at the EGPRN website https://www.egprn.org/page/conference-abstracts

**DOI:** 10.1080/13814788.2021.1879789

**Published:** 2021-02-23

## Introduction to theme of the conference: digital medicine and e-health

In October 2020, EGPRN welcomed researchers and clinicians interested in the primary care implementation of digital medicine and e-health. Digital medicine and e-health have the property of transcending time, place and human power symmetry issues. They cannot replace wisdom, intuition and bedside clinical judgement but with the growth of technological innovations, they increasingly add to these skills and competencies. Digital medicine and e-health are overlapping concepts about using technologies as tools for measurement and intervention in health care, including treatment, disease monitoring, disease prevention, and health promotion. E-health is healthcare practice supported by electronic processes and communication. It can also include health applications and links on mobile phones, referred to as mHealth or m-Health.

**KEYNOTE LECTURES**

## From remote sensors to digital health – A SWOT attempt

Eva Hummers

Department of General Practice, Universitätsmedizin Göttingen/University Medical Center Georg-August-Universität, Göttingen, Germany

**CONTACT** eva.hummers@med.uni-goettingen.de

SCREEN –AF is a randomised controlled diagnostic trial that recruited 856 patients from 48 family practices in Canada and Germany. An adhesive skin patch continuously recording an ECG for two weeks was used to screen for atrial fibrillation episodes in moderate- to high-risk patients and compared to usual care. The trial will be presented as an example of remote sensor use, which may be perceived as an entry to E-health in primary care.

Implementation and routine use of various E-health tools or applications vary widely across Europe. While use and access to connected electronic patient records by several care providers across healthcare levels, or patients themselves, is a matter of course in some countries, it may be considered quite revolutionary and threatening in others (for example data privacy or liability issues). The same holds for telemedicine approaches, electronic communication between GPs and patients, and the use of mobile devices or software ‘apps.’

Some GPs or other stakeholders may see opportunities for effective organisation of care, or better diagnosis and monitoring of health conditions, or more individualised treatments. Others are concerned that introducing more e-technology may eat away at person-centred, biopsychosocial care, or other family medicine key features.

Many patients like to consult ‘Dr Google’ before or along with their family doctor, even if some may have struggled to cope with the information obtained. Health-conscious citizens purchase wearable sensor devices, believing that ‘tracking’ health-related parameters will maintain or improve their health. Many share their data with the manufacturers of these devices or the Appendant software, or social media platforms. Others may face difficulties when required to administrate their health insurance or healthcare provider appointments online.

In parallel, the health and healthcare sector is a huge business opportunity for technology, software or smart service companies, who already invest massively into developing and marketing health-related products – and perceive a good return of investments.

From both a family doctor and a researcher perspective, it does not seem wise to ignore the brave new world of digital health. It certainly has weaknesses and threats. However, it also offers strengths and opportunities to family doctors and their patients, and certainly to researchers, who can discover, develop, and evaluate the new features of a digital culture against the background defined by the core characteristics of general practice/family medicine.

## Don’t get lost in translation of e-health to the real-world general practice!

Cecilia Björkelund

Primary Health Care, School of Public Health and Community Medicine, Institute of Medicine Sahlgrenska Academy University of Gothenburg, Gothenburg, Sweden

**CONTACT** cecilia.bjorkelund@allmed.gu.se

The e-health implementation must be developed to fit into the primary care and general practice context, and not the other way around. The development should be in cooperation, where general practice and the patients are the stakeholders and show the direction. The way to take a collective responsibility is to assert primary care and the users – our patients – already in the development and implementation phase of e-Health. General practice has a great responsibility to claim that an e-health treatment programme or a support programme – which most of e-health apps are – should be implemented in primary care by way of research activities. This could preferably be made as a method development programme, where the first part is a randomised controlled study performed in the primary care context. Many developers should object to that, arguing that this would take too long and cost too much. However, we need support for this claim of evidence, which is already an obvious demand for other parts of health care treatment and support methods. In Great Britain, NHS Digital has presented robust standards for developing safe software, apps and IT systems and deploying and operating such systems within the health and care environment. For general practice/primary care, effective internet-based treatment programmes and health apps are of great importance. However, its effectiveness must be evaluated in several ways: efficacy for the patient, usefulness for health care, and effectiveness for society. This means that, as general practitioners and health care workers, we must design studies that make both implementation and evaluation as effective as possible. Moreover, in all this strain to reach the highest effectiveness, we must never underestimate the utmost therapeutic effectiveness of the patient/person-centred consultation and continuity of care. In primary care/general practice, what we can and must do is to claim the importance of standards for the development and treatment of safe software, apps and IT systems and claim that these devices should be developed in the primary care context with primary care and users/patients leading the research and development within primary care.

## Primary care research networks: Why and how?

Guri Rørtveit

Department of Global Public Health and Primary Care, University of Bergen, Norway

**CONTACT** Guri.Rortveit@uib.no

The lack of clinical research in primary care is a problem that has been addressed by clinicians, researchers, health authorities and politicians alike. Clinical research in primary care is hard work, logistically. The researcher has to perform a two-step process; first recruit GPs for the study, and second support the clinician in recruiting patients. This cycle has to be done repeatedly for each patient and each study. For the clinicians, research invitations come unpredictably, and without advice from scientifically as well as clinically competent authorities. Each practice or even every GP must decide whether the research project has the necessary scientific standard or clinical relevance. Missing competence to assess this may result in a decline of participation due to uncertainty. In contrast, many clinicians may be interested in participating in research if a minimum framework is in place. Such a framework must include support for practical tasks, training, available time from other duties, reasonable funding and relevance for own practice.

The lack of framework in many places represents a waste of time for clinical researchers and a waste of resources for the society. Ultimately, it reduces the patients’ opportunity to participate in research of their interest. Primary Care Research Networks are infrastructures of clinical practices linked together by a research institution with employees who actively recruit clinicians to the network and help them stay ‘research ready.’ Furthermore, the network supports researchers in recruiting patients and obtaining data, which also reduces the burden on the clinician. Research networks already exist in the United Kingdom, the Netherlands, Ireland and other countries. In addition to supporting the obtaining of high-quality data with less effort for the researcher, they also support international collaboration. In Norway, we are currently establishing a nation-wide network. The vision for the Norwegian Primary Care Research Network is to support research of high quality that ultimately improves our patients’ health. Establishing research networks in primary care is an adequate response to current and future challenges in health care services.

## SELECTED ABSTRACTS

### THEME PRESENTATIONS

## Pioneering teledermatology in lithuanian primary care

Greta Petkeviciute, Roberta Lakstinyte, Kristina Ziuteliene, Ida Liseckiene and Leonas Valius

Faculty of Medicine, Lithuanian University of Health Sciences Kaunas Clinics Department of Family Medicine, Lithuanian College of Family Physicians, Lithuanian University of Health Sciences Medical Academy, Kaunas, Lithuania

**CONTACT** petkeviciutegret@gmail.com

**Background:** Teledermatology – the process of diagnosing dermatological problems, in the case of store-and-forward technology – is a new method of diagnosing skin conditions in Lithuanian health care. Also, it is a cost-effective tool to increase access of services to patients. Patients’ cost savings and benefits are especially pronounced in rural areas with long waiting times to see a dermatologist. This is the first project using store-and-forward technology to administrate teleservices in Lithuanian health care.

**Research question:** Are family medicine physicians (FMP) capable of providing teleservices? What are the differences between diagnosis in dermatologist and qualified FMP’s reports?

**Methods:** A prospective pilot study was performed from November 2018 till January 2020. Subjects were 152 patients visiting the Family Medicine department. They were consulted for a skin check-up by FMPs qualified in dermatoscopy and an advanced nurse practitioner. The store-and-forward technology managed patients’ information. Skin lesions were photo-documented using a digital dermatoscope. All data were sent to the dermatologist for consultation.

**Results:** The 152 patients included were aged between 11–88 years old. During the 55 FMP visits, 445 dermatoscopy images were taken. The most common diagnosis was seborrhoeic keratosis. Four malignant skin cancer cases (1 malignant melanoma, 1 squamous cell carcinoma, 2 basal cell carcinoma) were diagnosed and completely treated during this study. Consistency between FMPs diagnosis and dermatologists reported diagnosis was 92.4%. Only 7.6% of the diagnosis were not consistent between FMP and dermatologist. Forty-three patients (28.3%) were referred for additional treatment to the dermatologist. The maximum time from registration to seeing an FMP for a skin check-up was four days, compared to 14–28 days for a dermatologist appointment. The approximate time until the dermatologist report was completed was seven days.

**Conclusion:** FMPs qualified in dermatoscopy are capable of providing teleservices. Our pilot study suggests that store-and-forward teledermatology in FMP practice could lead to a 71.7% reduction in referrals to a specialist.

## Electronic health records across Europe: Adoption, digital maturity, and implications for quality and safety of care

Ana Luisa Nevesh, Ferdinando Petrazzuoli, Robert D. Hoffmann, Heidrun Lingner, Hans Thulesius and Jean-Yves Le Reste

Center for Health Technology and Services Research, University of Porto, Portugal; Institute of Global Health Innovation, Imperial College London, London, UK

**CONTACT** ana.luisa.neves@gmail.com

**Background:** Electronic health records (EHR) transform health services by providing new mechanisms for accessing personal medical records, submitting incident reports, and communicating across care settings. However, despite government efforts all over Europe, little is known about current adoption rates and digital maturity levels in different countries. Furthermore, despite the growing body of evidence on the theorised benefits of EHR on quality and safety of care, there is still a considerable gap between the predicted and demonstrated implications.

**Research question:** This work aims to characterise EHR use (adoption and digital maturity) by Primary Care Physicians (PCP) across Europe and evaluate their perspectives on the impact on the quality and safety of care.

**Methods:** The study used an online questionnaire survey of PCPs from 20 countries. Each national lead recruited PCPs through their contact networks directly by email, at a minimum number of 25 participants. The survey included multiple choice questions to characterise the system features and adoption rates. Digital maturity was evaluated using the “Patient-centred Framework for Evaluating Digital Maturity of Health Services”. Quantitative data was analysed using SPSS.

**Results:** A total of 1528 PCPs replied to the survey (60% female, 79% aged between 30 and 70 years). More than 60% of the respondents worked in an urban setting. More than 70% of the participants agreed on having achieved maturity in the dimensions ‘usage,’ ‘resources’ and ‘ability.’ Almost 60% agree that the EHR system has a positive impact on outcomes. However, less than 30% report having implemented digital evaluation methods in their practice.

**Conclusion:** EHR usage is far from universal, but we need to gather data on the current state of usage across Europe, as well as PCP beliefs regarding safety and quality of care. This data will allow us to inform health management personnel about the situation and needs.

## Predicting primary care physicians’ intentions to use e-health – A survey study based on the theory of planned behaviour

Veronica Milos Nymberg, Miriam Pikkemaat and Hans Thulesius

Clinical Sciences Malmö, Malmö, Sweden

**CONTACT** veronica.milos_nymberg@med.lu.se

**Background:** While e-health is remodelling health care worldwide, we know little about primary care physicians’ attitudes and expectations of e-health. Research about primary care physicians’ use of e-health, that is, digital contacts, digital tools or artificial intelligence (AI) may be useful for planning educational efforts and future implementation of digital technology in health care.

**Research question:** This study aimed to explore the experiences and behavioural intentions of Swedish primary care physicians towards e-health in primary care with a focus on behavioural predictors derived from the theory of planned behaviour.

**Methods:** We designed a web-based survey with a focus on attitudes, subjective norms and perceived behavioural control. The survey was sent to 1100 primary care physicians in two Swedish regions, from May to August 2019. Main outcome measures were scores for intentions to use e-health. Multiple regression analyses were made to study the correlation between predictors for using e-health derived from the theory of planned behaviour.

**Results:** Total response rate was 18%,198 returned surveys of which 134, 154, 161 and 171 respondents reported, respectively, no use of e-mail (68%), video consultations (78%), chat (81%), or SMS (86%) in their everyday clinical work. Yet, most respondents described positive intentions to use e-health in patient care for all three studied domains: digital contacts, chronic disease monitoring and AI. Attitudes and perceived behavioural control were significant predictors (*p* < 0.01) for intentions to use digital contacts (*R*^2^ = 0.54), monitoring disease with digital tools (*R*^2^ = 0.47) and AI (*R*^2^ = 0.54).

**Conclusion:** Swedish primary care physicians reported high behavioural intentions to utilise e-health. Attitudes, subjective norms and perceived behavioural control were strong predictors for using digital contacts. Social pressure translated into subjective norms was not correlated with intentions to use digital tools for chronic disease monitoring or AI, probably due to their current low use in primary care.

## Video consultations as an alternative to face-to-face consultations in primary care

Anthony Heymann, Inbal Moses and Ori Harel

University Tel Aviv, Tel Aviv, Israel

**CONTACT** tonyheymann@gmail.com

**Background:** Telemedicine is the use of communication networks for delivering health care services from one geographic location to another. It is a complex task that carries some inherent difficulties and risks compared to regular consultations. For six months, patients in the Meuhedet HMO have been able to book video consultations with their GP, who will then conduct the meeting while assessing the patient's electronic medical record. The GP can send the patients prescriptions or referrals electronically as required.

**Research question:** This study aimed to identify the characteristics and satisfaction of the video consultation users and identify the barriers and promoters to the use of the service by both patients and physicians.

**Methods:** This is a mixed-methods study including (1) data pertaining to the implementation of the video consultation, (2) a patient survey of their video consultation experience and (3) a focus group of physicians that participated in the pilot. Data from 26 physicians was collected over six months starting in May 2019.

**Results:** The patients seemed highly satisfied with their video visits (score 4.63/5.0). During this period, 2150 digital visits were scheduled,40% were completed. There were 20% no-show as opposed to 29% for regular visits, 33% were cancelled in advance by the patient. Average patient age was 32.8 years compared with 33.8 for regular visits. In 61% of video visits, patients were female compared with 54% for regular visits. The physicians in the focus group felt that the video service was a positive change, noting improved time management and better access for patients such as for mothers with young children. The medico-legal issue was not regarded as a barrier because physicians felt free to invite the patients for a regular visit if necessary.

**Conclusion:** Both physicians and patients were satisfied with the technology which enabled effective physician–patient interaction.

## Referral and hospital admission rates at prisons offering scheduled or unscheduled primary care and psychiatric video consultation

Katharina Schmalstieg-Bahr, Peter Merschitz, Joachim Szecsenyi, Eva Blozik and Martin Scherer

University Hospital Hamburg Eppendorf, Hamburg, Germany

**CONTACT** bahr@uke.de

**Background:** In comparison, prison inmates are at a higher risk for drug abuse, psychiatric or infectious diseases. Although intramural health has to be equivalent to extramural services, prison inmates have yet less access to specialised health care services. Often, transport to the nearest extramural medical facility is resource-intensive. Video consultations may offer the chance to deliver cost-effective health care for those patients.

**Research question:** How often and why are referrals to secondary care and hospital admissions needed when a scheduled or unscheduled video consultation is offered at a prison?

**Methods:** In five German prisons, a pilot project was conducted to assess feasibility, acceptance and consultation reasons of primary care and psychiatric video consultations between June and December 2018. This analysis includes the data of 436 consultations from June 2018 to February 2019 and focuses on referral and admission rates, as well as reasons.

**Results:** Most consultations were scheduled (341/436). In 67,4% (294/436) of all consultations, the patient was asked to come back if symptoms persisted or got worse. In 26,6% (116/436), a follow-up appointment with the video consultant or prison physician was scheduled. A referral to other specialties, most often psychiatry, was necessary in 3.9% (17/436) cases. Only 1.8% (8/436) needed a hospital admission. Usually, (7/8) an admission resulted from an unscheduled consultation and video was used in 87% (7/8). Reasons for admissions were severe abdominal pain, hypotension, unstable angina/suspected myocardial infarction or a suspected schizophrenic episode.

**Conclusion:** Most scheduled and unscheduled consultations did not require subsequent patient transport to external healthcare providers. Using telemedicine allowed a prompt consultation with the possibility to refer patients to other specialties or hospitalise them when necessary.

## (Digital) communication between general practitioners and nursing homes in Germany 2019

Eric Kroeber, Thomas Frese and Alexander Bauer

Institute of General Practice and Family Medicine, Martin-Luther-University Halle-Wittenberg, Halle, Germany

**CONTACT** eric.kroeber@posteo.de

**Background:** The German health care system is facing a sizable challenge shortly. Currently, almost one million care recipients are living in nursing homes. There is a shortage on caregivers and General Practitioners (GPs).

**Research question:** This study investigates basic information on communication routines and difficulties as well as GP’s perspectives on E-health-technologies in patient care in nursing homes.

**Methods:** A questionnaire-based cross-sectional study, carried out among *n* = 600 randomly selected GPs in Germany sent by mail.

**Results:** The response rate was 19.8% (*n* = 114). The respondent’s mean age was 53 years (min =35; max:0 = 77), two thirds were women (65.1%). GPs commonly use fax (92.3%) and telephone (86.5%) to communicate with nursing homes. Less than 10% routinely use e-mail (6.2%), e-health software (5.3%) or chat-services (0.9%). About half of GPs regard unnecessary (52.3%) or unspecific (50.5%) nursing home visit requests as well as medication plan changes by other physicians (51.4%) as a common but evitable problem. Many GPs want to use e-medication plans (84.6%), e-follow-up prescriptions (78.8%) and e-letters of referral (69.2%) in the future. 32.7% of GPs already have fully digitalised patient files. Only 8.7% work exclusively paper-based.

**Conclusion:** Though GPs are open for digitalised communication with nursing homes, fax and telephone are still mostly used. GPs prefer to execute fewer complex tasks digitally, like changes of medication plans and letters of referral. Fewer can imagine digital solutions for complex procedures like acute health problems and ward rounds. Only 7.6% do not want to work digitally at all.

## Digital therapeutics: Technology innovation to face general practice’s challenges in 2020

Alberto Malva, Francesco Del Zotti and Giuseppe Recchia

Fondazione Smith Kline, Verona, Italy

**CONTACT** albertomalva@outlook.com

**Background:** Digital therapeutics (DTx) are evidence-based software aimed to prevent, manage, or treat a broad spectrum of physical, mental and behavioural conditions. These interventions are typically subject to regulatory oversight and can be standalone or in combination with traditional drug therapies. DTx forms an independent category of evidence-based products within the broader digital health landscape and are distinct from pureplay adherence, diagnostic, telehealth products.

**Research question:** Here, we provide a review of current evidences and challenges regarding the possible use of DTx in family medicine practice.

**Methods:** Review until June 2019 of the DTx suitable for family medicine approved/under approval by FDA or under development according to the top 10 tech start-ups listed in order of funding.

**Results:** reSET® is the first DTx approved by FDA (2017) as cognitive behavioural therapy (CBT) for the treatment of substance use disorder – abstinence rate improvement versus human performed CBT 40.3% vs 17.6%. Respimat® combines software and hardware program to improve asthma and COPD control and optimise healthcare utilisation. Dthera Sciences delivers reminiscence therapy to Alzheimer’s patients in a scalable and personalised manner. Spleepio™ performs sleep improvement program featuring CBT techniques. KAIA delivers physical exercises and behavioural therapy for chronic back pain patients. Several companies have developed software which engages patients with type-2 diabetes, hypertension and obesity to improve self-management and outcomes.

**Conclusion:** DTx has significant potential to affect primary care landscape thanks to empowering patients, healthcare providers and payers through intelligent and accessible tolls for addressing a wide range of conditions *via* data-driven interventions. The role of GPs in the research, development and delivery of DTx is crucial but still to be determined as well as the contribution expected from European GPs to adapt and delivery DTx developed in the US concerning regulatory, social and ethical point of view.

## FREE PRESENTATIONS

## A prospective observational cross-sectional study with focused cardiac ultrasound (FOCUS) conducted by the family physicians at patients with a high risk of cardiovascular diseases.

Mihai Iacob

Department of Research in Primary Health Care, Ultrasound Working Group in Family Medicine - AEDUS/EUVEKUS, Timisoara, Romania

**CONTACT** dr_iacob@yahoo.com

**Background:** FOCUS is a complement of the clinical examination, evaluating the structural and functional abnormalities of the heart, to the critical hemodynamic patient. A few studies have assessed the value and accuracy of focussed cardiac ultrasound (FOCUS) performed by family physicians. This study aimed to evaluate the diagnostic accuracy of FOCUS performed by family doctors compared to echocardiography performed by a cardiologist at the patients with a high risk of cardiovascular diseases.

**Research question:** How can we improve the rapid evaluation of the critical hemodynamic patient in primary healthcare?

**Methods:** We performed FOCUS on patients who presented suspicion of cardiac pathology (cardiomegaly, valvulopathy, pericarditis, endocarditis, congenital malformations, aneurysms, and arrhythmias) after clinical examination and used the fivestandard cardiac scans: Subxiphoid-view, Parasternal-long/short axis, Apical-four-chamber-view, and IVCassessment. We conducted a prospective-observational-cross-sectional-study of 1780 patients with high cardiovascular risk. High-risk patients identified on inclusion criteria, were first examined by a family doctor with expertise, subsequently compared with ultrasound review by cardiologists, to determine this application’s accuracy. We have developed a Computerised Diagnostic Algorithm of the cardiac-pathology detected by non-cardiologists. The agreement between family physicians and cardiologists on each finding was evaluated using Cohen’s kappa coefficient with 95%CI.

**Results:** We identified 585 patients with cardiac-pathology who were subsequently confirmed by the cardiologist. We completed a descriptive-statistical-analysis of the echocardiographic cases detected. The accuracy of FOCUS-screening in primary care was 96.07% with a sensitivity: 95.12% and specificity: 96.57%, *p* < 0.001, for all 1780 emergency-patients who were subsequently confirmed by the cardiologist as the ‘Gold-Standard’ method. The prevalence of cardiac pathology was 34.55% with 95%CI: 32.34–36.81%. Reports of the two-groups for identifying cardiac-pathology showed a 95%-agreement (*k* = 0.88; 95%CI =0.81–0.95), standard error: 0.037.

**Conclusion:** FOCUS performed by trained family physicians is comparable to echocardiography performed by cardiologists. It could be a reliable tool and screening test for the initial diagnosis of patients suspected of cardiac-abnormalities. We propose FOCUS as a complementary-diagnostic tool followed by referral to the cardiologist.

## How GPs decide to assess cardiovascular risk factors in European countries – a qualitative EGPRN fellowship study

Ilze Skuja, Aleksandra Trubovich, Emilie Femtehjell, Marta Marques, Elina Skuja, Aivars Lejnieks and Michael Harris

Riga Stradins University Ilze Skuja GP Practice, Family Medicine, Riga, Latvia

**CONTACT** skujailzedr@gmail.com

**Background:** Cardiovascular disease (CVD) is the main cause of death in Europe, leading to 3.9 million deaths a year (45% off all deaths). General Practitioners (GPs) are often the first clinicians to be consulted by patients. They need to assess CVD risk factors (RF) and initiate preventive measures hence overall prevalence of CVD morbidity and mortality can be reduced.

**Research question:** How do GPs think and act when seeing patients who might have CVD RFs?

**Methods:** A semi-structured questionnaire was designed following a literature review. Researchers used this to interview GPs in five countries (Latvia, Portugal, Norway, Russia and England) until data saturation. Data were transcribed, translated into English, coded, validated, then divided into themes and sub-themes, with comparisons made across the participating countries.

**Results:** We collected ten interviews in Latvia, Russia, Norway and Portugal and eight interviews in England, each lasting 25–45 min. Relevant differences between national health care systems included GPs’ teams structure and their financial support. We identified eight overarching themes relating to how GPs assess CVD risk: ranking of the relative importance of different RFs; indications to perform RF assessments; typical profiles of patients that need RF assessment; the effect of obesity; use of guidelines; assessment using SCORE risk charts; limitations and problems associated with CVD risk assessment.

**Conclusion:** While GPs’ knowledge of CVD RFs is broadly similar across these countries, there are national variations in practitioners’ approaches. These differences are driven by variations in financial, historical and geographical factors, as well as specific differences in GPs’ knowledge. However, we found more similarities than differences between the views of GPs in the five countries studied, and these may provide a basis for a Europe-wide primary care approach to acting on CVD risk factors.

## Perceptions and experiences of lung cancer patients regarding collaboration between general practices and hospitals: Results of a qualitative interview study

Jasmin Bossert, Katja Krug, Johanna Forstner, Michael Thomas, Matthias Villalobos, Anja Siegle, Corinna Jung, Nicole Deis and Michel Wensing

Department of General Practice and Health Service Research, Heidelberg, Germany

**CONTACT** Jasmin.Bossert@med.uniheidelberg.de

**Background:** Lung cancer patients with comorbidity often require treatment and care by different health professionals, in various settings, and at different points in time during the course of the disease. To organise and coordinate healthcare efficiently, effective collaboration between general practitioners and hospitals is required.

**Research question:** This study aimed to assess the views of advanced lung cancer patients with comorbidity regarding coordination of care across healthcare sectors.

**Methods:** This qualitative study used face-to-face guide-based semi-structured interviews with advanced lung cancer patients and their informal caregivers. Data were audio-recorded, pseudonymised and transcribed verbatim. Data analysis was performed using Qualitative Content Analysis to structure data into themes and subthemes.

**Results:** In 15 interviews, participants reported that cross-sectoral collaboration functioned well if treatments were carried out as planned. Whenever treatment gaps did occur, participants assumed the cause to be different levels of information among general practices and hospitals. General practitioners are often not informed about changes in medication and treatment regimens during the hospital stay. As a result, participants felt to take responsibility for the coordination of care. However, they perceive a general practitioner should assume this role. Potential for optimisation of cross-sectoral collaboration was seen in the way of communication by using an electronic platform.

**Conclusion:** A more intensive exchange *via* an electronic platform was perceived to support the general practitioners’ information level about hospitalisation. Despite growing evidence that patient-managed platforms can have positive benefits for healthcare, such concepts are not yet widely adopted in Germany. Therefore, pressure is growing to address barriers for implementing digital information transfer, which could have major implications for the coordination of health care in general practice.

## Is there a relationship between adherence to medications and adherence to preventive medicine?

Michal Shani, Alex Lustman, Yochai Schonmann and Doron Comaneshter

Family Medicine, Clalit Health Service, Mazkekret Batya, Israel

**CONTACT** michal.shani@gmail.com

**Background:** Medication adherence is crucial in chronic patients’ care. Better medication adherence is related to a better outcome. The association between medication adherence and adherence to preventive medicine has not been tested.

**Research question:** Is there a relation between adherence to medications and adherence to preventive medicine?

**Methods:** We included all patients aged 50–75 with diabetes or hypertension insured by Clalit and treated with at least one chronic medication in 2017. For each patient, we examined the adherence to one of 22 oral medications for the treatment of diabetes or hypertension. Good adherence was defined as claiming at least nine monthly prescriptions during 2017. We calculated for each patient the average adherence rate for her/his medications. We tested the relation between average medications adherence rate and whether patients had annual influenza injections, mammogram and colon cancer screen according to the recommendations.

**Results:** Overall, 262,649 patients were included. Average age was 63.7, 50.6% were men. 81.5% of the patients had hypertension and 59.4% had diabetes. Patients used 2.2 ± 1.1 medications on average. Eligible patients (59.6%) had received an influenza vaccine during 2017, 67.8% had undergone colon cancer screening, and 75.1% of the women had mammography according to the recommendations. Patients who received an influenza vaccine had higher adherence rates to medication compared to patients who did not have the injection OR =1.27 (CI 1.25–1.30), patients who performed mammography had OR =1.15 (CI 1.11–1.18) for medication adherence rate compared to those who had not, and patients who had been screened for colon cancer had OR =1.18 (CI 1.16–1.21) for medication adherence compared to those who had not.

**Conclusion:** Our findings suggest that medication adherence is associated with adherence to preventive medicine in diabetic and hypertensive patients.

## Development and validation of a tool assessing knowledge and attitudes regarding adult vaccination: The attitude towards adult VACcination (ATAVAC) questionnaire

Philippe-Richard Domeyer, Dimitrios Gougourelas, Konstantinos Kolokas, Anastasia Papaioannou, Vasileios Gkizlis, Emmanuil Chatzimanolis, Sofia Birka, Ioanna Tsiligianni, Athina Tatsioni and Zoi Tsimtsiou

**CONTACT** philip.domeyer@gmail.com

**Background:** Despite the unequivocal value of vaccination in reducing the global burden of infectious disease, antivaccination movement thrives. To our knowledge, no fully validated tool exploring knowledge and attitudes of primary care patients regarding adult vaccination exists.

**Research question:** Our study aimed to develop and validate a questionnaire assessing knowledge and attitudes regarding adult immunisation.

**Methods:** This national cross-sectional study included 2070 adult patients who presented for routine care in 23 Greek public Primary Healthcare Units. The development of the questionnaire was a result of literature review, semi-structured interviews and pilot-testing of its preliminary versions to researchers and patients. The questionnaire’s initial version contained 15 items measuring the respondents’ knowledge and attitude towards adult immunisation on a six-point Likert scale. The sample was randomly split into two halves. Exploratory factor analysis, performed in the first sample, was used to create multi-item scales; confirmatory factor analysis was used in the second sample to assess goodness of fit.

**Results:** The final sample consisted of 1571 individuals. Overall, Cronbach’s alpha was 0.844. The initial exploratory factor analysis resulted in a three-factor model. The subsequent confirmatory factor analysis indicated that an 11-item version of the scale provided the best fit of the model to the data (root mean square error of approximation, RMSEA =0.050; comparative fit index, CFI =0.955, Tucker Lewis index, TLI =0.937; standardised root mean square residual, SRMR =0.053).

**Conclusion:** The ATAVAC instrument proved to be a reliable and valid tool, suitable for assessing knowledge and attitudes regarding adult vaccination.

## Potential correlates of burnout among general practitioners and residents in Hungary: The significant role of gender, age, dependent care and experience

Peter Torzsa, Andras Mohos, Csenge Hargittay, Bernadett Markus, Laszlo Kalabay and Szilvia Adam

Department of Family Medicine, Semmelweis Egyetem, Budapest, Hungary

**CONTACT** torzsa.peter@med.semmelweis-univ.hu

**Background:** Burnout is increasingly prevalent among general practitioners (GPs) in Hungary, leading to functional impairment and, subsequently, to poor quality of patient care. However, little is known about potential predictors of burnout among GPs.

**Research question:** What are the psychosocial correlates of burnout among GPs and residents in Hungary?

**Methods:** We collected socio-demographic and work-related data with self-administered questionnaires in a cross-sectional study among GPs (*N* = 196) and residents (*N* = 154). We assessed burnout with the Maslach Burnout Inventory Human Services Survey (MBI-HSS) and calculated the mean level of burnout and the proportion of physicians suffering from low, intermediate and high degree of burnout. We deployed Mann–Whitney U-test to explore gender disparity in the level of burnout between female and male physicians and between general practitioners and residents.

**Results:** The prevalence of moderate to high-level emotional exhaustion, depersonalisation, and impaired personal accomplishment was 34.7%, 33.5% and 67.8% as well as 41.0%, 43.1%, and 71.1% among GPs and residents, respectively. Residents reported significantly lower level of personal accomplishment versus GPs. We identified a significantly higher level of depersonalisation among male physicians compared to female physicians. Age correlated negatively with emotional exhaustion and depersonalisation and positively with personal accomplishment among GPs. Dependent care was positively associated with burnout among female GPs. Female residents were more likely to report depersonalisation. High workload was positively correlated with depersonalisation among female GPs. Younger age emerged as the strongest predictor of emotional exhaustion. Male gender and fewer years of experience predicted depersonalisation best, and male gender showed a significant predictive relationship with low personal accomplishment.

**Conclusion:** We identified specific socio-demographic and work-related correlates of burnout, which may guide the development of specific and effective organisational decisions to attenuate occupational stress and subsequent burnout as well as functional impairment among GPs, and thus, may improve the quality of patient care.

## Health status and social support in long-term cancer survivors: A cross-sectional study

Magdalena Esteva Cantó, Beatriz Leon, Joan Llobera, Marc Casajoana, Edurne Zabaleta- Del-Olmo, Tomás López-Jiménez and Bonaventura Bolibar

Majorca Primary Care District, Palma de Mallorca, Spain

**CONTACT** mesteva@ibsalut.caib.es

**Background:** Quality of life and social support are key elements during the cancer survivorship period.

**Research question:** Do cancer survivors display a worse health status and less social support than those who have not suffered a cancer?

**Methods:** Descriptive study nested in phase II and III of a cluster randomised trial (EIRA study) to establish the effect of a complex intervention individual, group and community) in primary care that aims to decrease tobacco consumption, the low adherence to a Mediterranean diet and low physical activity. Setting: 38 health centres of 11 provinces of Spain. Participants: Subjects 45–75 years old with almost two risky behaviours were included. Measurements: Sociodemographic, diet, physical activity, tobacco consumption, body mass index, Charlson comorbidity index, diagnosis of cancer, health status perception and social support.

**Results:** We included 4259 patients; 190 (4.46%) were cancer survivors. Cancer survivors were older (62.8; SD =7) compared to non-cancer persons 58.7 years; (SD =8; *p* < 0.01). Prevalence of permanent disability was higher in cancer survivors 11.9% vs. 3.5%; *p* < 0.001). No differences were observed between groups in smoking, adherence to Mediterranean diet and physical activity, obesity and scores of social support. Cancer survivor patients perceived their health status lower than non-cancer persons (OR 1.82 CI95% 1.02–2.75), had higher percentage more than 1 health problems (OR 1.68 95%CI 1.18–2.39), higher presence of COPD (OR 2.17; 95%CI 1.25–3.78), and depression (OR 1.65; 95%CI 1.06–2.57). Adjusted model shows a higher risk of worse perception of health status and age.

**Conclusion:** Cancer survivors suffer from a higher number of chronic diseases, have more permanent disability and value their health status as bad with more frequency than people without cancer but social support is similar in both groups. More than 80% of cancer survivor’s maintain unhealthy behaviour in high proportion. This could represent an important problem for their future health and quality of life.

## Erroneous computer-based ECG interpretations of atrial fibrillation and atrial flutter in a Swedish primary health care setting

Hans Thulesius, Thomas Lindow, Erik Ljungström, Josefine Kron and Olle Pahlm

Clinical Sciences, R&D Kronoberg, Växjö, Sweden

**CONTACT** hansthulesius@gmail.com

**Background:** Patients with atrial fibrillation and atrial flutter are common in primary care but data regarding the incidence of misdiagnoses in primary care settings are lacking. We aimed to describe the incidence of incorrect computerised ECG interpretations of atrial fibrillation or atrial flutter in a Swedish primary care population, the rate of correction of computer misinterpretations and the consequences of misdiagnosis.

**Research question:** What is the incidence of misdiagnosis of atrial fibrillation and atrial flutter in a computer-based ECG assessment system in primary care? And what is the impact of misdiagnosis?

**Methods:** We included all adult patients who had an ECG recorded at most primary health care centres in Region Kronoberg, Sweden, between January 2016 and June 2016 with a computer statement including the words ‘atrial fibrillation’ or ‘atrial flutter.’ Retrospective expert re-analysis of ECGs with a computer-suggested diagnosis of atrial fibrillation or atrial flutter was performed.

**Results:** Of 988 ECGs with a computer diagnosis of atrial fibrillation or atrial flutter, 89 (9%) were incorrect, among which the interpreting physician did not correct 36. In 12 cases, misdiagnosed atrial fibrillation/flutter led to inappropriate treatment with anticoagulant therapy. More atrial flutters, 27 out of 80 (34%), than atrial fibrillations, 62 out of 908 (7%), were incorrectly diagnosed by the computer.

**Conclusion:** In almost thousand consecutive ECGs with a computer-based diagnosis of atrial fibrillation or atrial flutter in a Swedish primary care setting the diagnosis was incorrect in one out of eleven patients and in almost half of these cases the misdiagnosis was not corrected by the interpreting primary care physician. Twelve patients received inappropriate anticoagulant treatment as a result of misdiagnosis.

## What are the factors that have hindered the achievement of the advance directives since the 2016 French law? A thematic analysis based on the grey literature review

Zambonino Marine, Le Goff Delphine, Collin Antoine, Nabbe Patrice, Hazif-Thomas Cyril and Jean Yves Le Reste

Brest, Brest, France

**CONTACT** marinezambonino@orange.fr

**Background:** Advance directives (AD) are a document giving medical instructions if the patient is no longer able to express them. This system was updated, in France, by the Claeys-Leonetti law in February 2016. Nevertheless, few French patients have a written AD. No data are available in the medical literature. What are or could be the factors hindering the achievement of AD since the Claeys-Leonetti law in grey literature?

**Research question:** What factors have hindered the achievement of the advance directives since the 2016 French law? Thematic analysis based on the grey literature review used.

**Methods:** Systematic literature review was undertaken between the 1 January 2016 and 31 December 2017 using the adapted PRISMA guide on Google Scholar. Search equation was using ‘advance directive,’ ‘community,’ ‘ethics’ as keywords. Exclusion criteria were: no reference to the French law, anticipated directives were not the main topics, the community was not mentioned, ethics was not mentioned. Inclusion criteria corresponded to the identification of hindering factors for the achievement of ADs. Documents were compiled using thematic and hermeneutic analyses. A heuristic map was created. All of the PRISMA process and coding were achieved with two researchers working blindly.

**Results:** In total 139 documents were found, 19 were eligible, 16 were included in the thematic analyses comprised six book extracts, one book, one study report, five publications, a dissertation and a thesis manuscript. A total of 101 axial codes were identified and grouped into 19 sub-themes assembled into 11 main themes: Individual sphere, care sphere, communication, temporality, opposability, interpretation, elaboration, psychic and spirituality, law and society.

**Conclusion:** One of the obstacles is the opposability of the AD. Many other limiting factors exist in legal, ethical, sociological, and practical affairs. They concern all actors implied in the AD, from the individual to the social groups. Education of health professionals about AD should be promoted to override these constraints.

## The effect of breast cancer on control of diabetes

Alexandra Verzhbitsky and Sofia Eilat-Tsanani

Department of Family Medicine, Bar Ilan University; Clalit Health Services, Afula, Israel

**CONTACT** alexaverba@gmail.com

**Background:** Diabetes mellitus (DM) is characterised by various long-term complications, between which association with malignant disorders, including breast cancer (BC). The contribution of DM to the development of BC has been discussed widely. In contrast, the breast cancer’s effect on the management of DM and the control of the disease have not been reported.

**Research question:** How does BC influence the control on DM?

**Methods:** A cross sectional study. Women with DM who were diagnosed with BC were included. Demographic data, information related to control of DM, date of onset of BC and related treatments were retrieved from the database of CHS. Control of DM was defined as HBA1C < 7.5% for women younger than 65 and <8.0 for women aged 65 years and older. Control of DM was evaluated 2 years before and after breast cancer diagnosis, considering demographic and morbidity factors. The study population included 220 women with DM who were diagnosed with breast cancer during 2005–2016, 56% were Jews. Obesity was recorded in 78%. Anxiety was diagnosed in 55% and depression in 43%. Surgical procedures were documented in 87% of the women, radiation in 67%. Oncologic medications were purchased by 31%. The trend towards controlled DM was reported in 186 women and towards not controlled in 30 women. According the logistic regression model the factors identified with chance for trend towards controlled DM were: for Arabs less than Jews [OR =0.2, 95% CI 0.09–0.7, *p* = 0.007), older age [OR =1.1, 95%CI 1.06–1.18, *p* < 0.001] not having mental disorder [OR =0.18, 95% CI 0.06–0.54, *p* = 0.002]

**Conclusion:** On average diagnosis of BC did not disrupt the control of DM. BC may raise the level of self-care. We should focus on younger women, Arabs and those having mental comorbidity to prevent deterioration of DM.

